# Enhancement of the Physical Stability of Amorphous Sildenafil in a Binary Mixture, with either a Plasticizing or Antiplasticizing Compound

**DOI:** 10.3390/pharmaceutics12050460

**Published:** 2020-05-18

**Authors:** Justyna Knapik-Kowalczuk, Krzysztof Chmiel, Justyna Pacułt, Klaudia Bialek, Lidia Tajber, Marian Paluch

**Affiliations:** 1 Institute of Physics, Faculty of Science and Technology University of Silesia, SMCEBI, 75 Pułku Piechoty 1a, 41-500 Chorzów, Poland; justyna.knapik-kowalczuk@us.edu.pl (J.K.-K.); justyna.pacult@smcebi.edu.pl (J.P.); marian.paluch@us.edu.pl (M.P.); 2 School of Pharmacy and Pharmaceutical Sciences, Trinity College Dublin, Dublin 2, Ireland; bialekk@tcd.ie (K.B.); ltajber@tcd.ie (L.T.)

**Keywords:** ASD, dielectric spectroscopy, physical stability, sildenafil, plasticizing effect

## Abstract

The main purpose of this paper was to evaluate the impact of both high- and low-T_g_ polymer additives on the physical stability of an amorphous drug, sildenafil (SIL). The molecular mobility of neat amorphous SIL was strongly affected by the polymeric excipients used (Kollidon VA64 (KVA) and poly(vinylacetate) (PVAc)). The addition of KVA slowed down the molecular dynamics of amorphous SIL (antiplasticizing effect), however, the addition of PVAc accelerated the molecular motions of the neat drug (plasticizing effect). Therefore, in order to properly assess the effect of the polymer on the physical stability of SIL, the amorphous samples at both: isothermal (at constant temperature—353 K) and isochronal (at constant relaxation time—τ_α_ = 1.5 ms) conditions were compared. Our studies showed that KVA suppressed the recrystallization of amorphous SIL more efficiently than PVAc. KVA improved the physical stability of the amorphous drug, regardless of the chosen concentration. On the other hand, in the case of PVAc, a low polymer content (i.e., 25 wt.%) destabilized amorphous SIL, when stored at 353 K. Nevertheless, at high concentrations of this excipient (i.e., 75 wt.%), its effect on the amorphous pharmaceutical seemed to be the opposite. Therefore, above a certain concentration, the PVAc presence no longer accelerates the SIL recrystallization process, but inhibits it.

## 1. Introduction

A growing interest in the advantages of amorphous drugs, such as solubility and dissolution rate in the water [1,2,3,4,5,6,7], over their crystalline counterparts resulted in numerous attempts to find preparative methods of amorphous pharmaceutical solids. However, the active pharmaceutical ingredients (APIs) in the amorphous form are thermodynamically unstable, and during the required shelf-life time, most of them return to their more stable, crystalline form [8,9,10,11,12]. Due to this fact, the use of inhibitors of crystallization is inevitable. Successful stabilizers can be categorized into two groups. Stabilizers of the first category, consisting of low molecular weight compounds such as: acetylated sugars [9,13], organic acids [14,15,16], or even different drugs [17,18,19,20,21,22], have proved to be very effective in inhibiting recrystallization. However, the vast majority of stabilizers are large molecular weight compounds (the second group), such as polymers [5,7,23,24,25,26,27,28]. The main reason for this is the variety of the possible applications of the latter. Focusing on a few of the features, the polymers can: i) affect the processing conditions (considering extrusion-based drug formulations) [28,29,30,31], ii) enhance water solubility [32,33,34,35,36,37] or, as stated above, iii) improve the physical stability of the APIs in the amorphous form [38,39,40].

The polymer’s ability to improve the physical stability of the amorphous pharmaceuticals is not fully understood, although a few factors affecting this property have been well described in the literature. Firstly, the ability to form intermolecular interactions between the drug and the polymer (such as ionic interaction, dipole–dipole interactions or hydrogen bonding) is one of the crucial factors to consider when preparing a stable amorphous solid dispersion (ASD) system [40,41,42,43]. Secondly, the addition of a polymer with a high glass transition temperature (T_g_) (well above the T_g_ of the amorphous API) will increase the T_g_ of the system—the so-called antiplasticization effect—which corresponds to the deceleration of the molecular mobility controlling the cold crystallization process at a certain temperature [44,45]. In contrast to the above, a polymer with a low T_g_ (well below the T_g_ of the amorphous l) will decrease the T_g_ of the system—the so-called plasticization effect—which corresponds to the acceleration of the molecular mobility. It has been demonstrated, using the example of a binary mixture of the model drug, indomethacin, with a polymer, poly(ethylene oxide) (PEO), that the plasticization effect can, indeed, accelerate its crystallization [46].

However, the overall effect on the drug’s physical stability exerted by the polymer addition is not universal. Namely, as it has been reported, the addition of the polymer with a high-T_g_ does not guarantee the enhancement of the physical stability [41]. Furthermore, a few reports confirmed the enhancement of the physical stability associated with the plasticization effect exerted by the used additive [18,47,48].

Therefore, the main purpose of this work was to evaluate the impact of both high- and low-Tg polymer additives, Kollidon VA64 (KVA) and poly(vinylacetate) (PVAc), respectively, on the physical stability of an amorphous API. Throughout this study, we compared the stability of the KVA and PVAc based ASD systems at both isothermal (at constant temperature) and isochronal (at constant relaxation time) conditions. In order to facilitate the investigations, we decided to perform the studies on sildenafil (SIL), of which the molecular dynamics, as well as the tendency towards recrystallization, have already been well-characterized in the literature [49,50]. The thermal properties of drug-polymer systems containing different concentrations of SIL and KVA or PVAc were investigated via differential scanning calorimetry (DSC). On the basis of DSC data, we determined the concentration dependencies of the T_g_s of SIL + KVA and SIL + PVAc ASD systems. To examine the molecular mobility of the investigated drug-polymer mixtures, we employed broadband dielectric spectroscopy (BDS). Furthermore, long-term crystallization studies were performed via BDS. The results allowed us to determine which polymer: KVA or PVAc, is the most effective inhibitor of crystallization of the investigated amorphous drug when stored at isothermal and isochronal conditions.

## 2. Materials and Methods

### 2.1. Materials

Sildenafil (SIL) with a molecular mass of M_W_ = 474.57 g mol^−1^ and purity ≥ 99% was obtained from Polpharma (Starogard Gdański, Poland). Kollidon VA64—vinylpyrrolidone-vinyl acetate copolymer— (KVA) with a molecular mass of M_W_ = 45,000–47,000 g mol^−1^ was purchased from BASF SE (Ludwigshafen, Germany). Poly vinylacetate (PVAc), with a molecular mass of M_W_ = 50,000 g mol^−1^, was purchased from Alfa Aesar (Karlsruhe, Germany). Infrared quality potassium bromide (KBr) was obtained from Honeywell (Offenbach am Main, Germany). All chemicals were used as received.

### 2.2. Preparation of Binary System

The sildenafil-based amorphous solid dispersion systems were prepared at different weight concentrations of KVA or PVAc. To acquire homogeneous samples, SIL was mixed with polymer at appropriate ratios in a mortar for approximately 20–30 min. In case of calorimetric measurements, the mixtures prepared in this way were then melted at T = 470 K, during the first DSC scan, and vitrified by fast cooling (20 K/min) during the second DSC scan. Sample preparation for the BDS measurements involved melting the binary physical mixtures at T = 470 K, followed by vitrification on a previously chilled copper plate. All measurements were performed immediately after preparation of the amorphous systems, to avoid recrystallization.

### 2.3. Differential Scanning Calorimetry

The thermodynamic properties of SIL, KVA, PVAc and their binary systems were examined using a Mettler–Toledo DSC 1 STAR^e^ System (Columbus, OH, USA). The measuring device was equipped with a HSS8 ceramic sensor with 120 thermocouples. The instrument was calibrated for temperature and enthalpy using indium and zinc standards. Crystallization and melting points were determined as the onset of the peak, whereas the glass transition temperature was measured as the midpoint of the heat capacity increment. The samples were measured in aluminum crucibles (40 µL). Measurements were carried out in the temperature range from 320 to 475 K for the KVA based systems and from 300 to 475 K for the PVAc based systems, with 10 K/min heating rate.

### 2.4. Broadband Dielectric Spectroscopy

The dielectric measurements of SIL-based ASDs were carried out using a Novocontrol GMBH Alpha dielectric spectrometer (Montabaur, Germany), in the frequency range from 10^−1^ to 10^6^ Hz at temperatures from 153 to 473 K, with a heating rate equal to 0.5 K/min. The temperature was controlled by a Quatro temperature controller, with temperature stability better than 0.1 K. Measurements of the dielectric permittivity at 1 kHz frequency were performed under nitrogen atmosphere in the temperature range from 270 to 450 K, with a heating rate equal to 2 K/min. Dielectric studies of SIL and its binary systems were performed immediately after vitrification by fast cooling of the melt in a parallel-plate cell made of stainless steel (diameter 15 mm, and a 0.1 mm gap with quartz spacers).

### 2.5. Infrared Spectroscopy

Infrared spectra were recorded on a PerkinElmer Spectrum 100 FT-IR Spectrometer (Shelton, Connecticut, United States) set up in the transmission mode using KBr pellets. The pellets comprised 1% w/w of the investigated compound dispersed in KBr and were prepared by applying 800 psi pressure in a level press. Each spectrum was scanned in the range of 600–4000 cm^−1^ with a resolution of 1 cm^−1^ and a minimum of 40 scans were collected and averaged. The spectra were then normalized and background corrected [51]. An infrared analysis was carried out on crystalline as well as amorphous SIL and SIL-based ASD samples, which were first melted at 468 K and then quenched.

## 3. Results and Discussion

### 3.1. Thermal Properties of SIL-Based Mixtures

**Figure 1:**
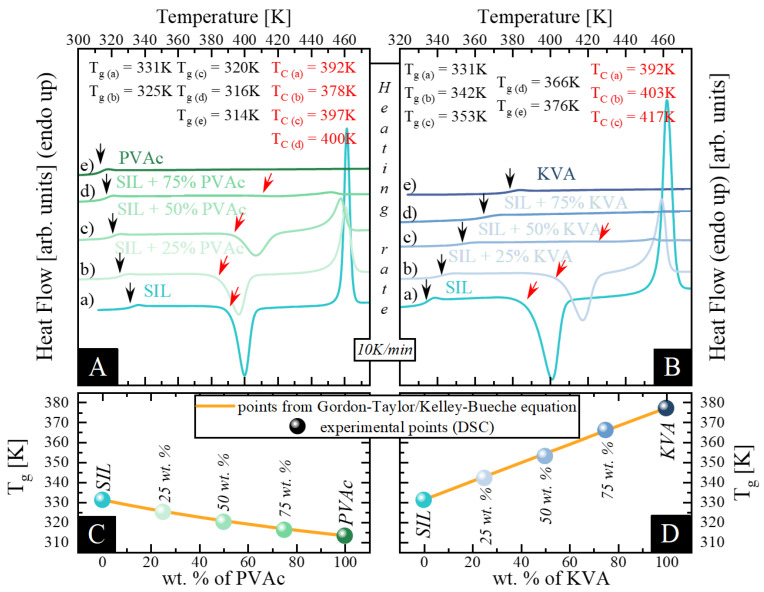
(**A**) shows thermograms of amorphous: a) sildenafil (SIL) (aqua), b) SIL + 25 wt.% poly(vinylacetate) (PVAc) (sage), c) SIL + 50 wt.% PVAc (mint), d) SIL + 75 wt.% PVAc (emerald) and e) PVAc (olive). (**B**) presents thermograms of amorphous: a) SIL (aqua), b) SIL + 25 wt% KVA (Kollidon VA64) (celeste blue), c) SIL + 50 wt.% KVA (baby blue), d) SIL + 75 wt.% KVA (cornflower) and e) KVA (royal). (**C**,**D**) present the glass transition temperatures of SIL–VAc and SIL–KVA mixtures respectively. Points correspond to the experimentally determined T_g_ values. The orange line represents the prediction by the Gordon–Taylor equation.

Thermal properties of neat components (amorphous SIL and KVA and PVAc polymers) and their binary amorphous mixtures, in the form of amorphous solid dispersion (ASD) systems (SIL + 25 wt.% KVA, SIL + 50 wt.% KVA, SIL + 75 wt.% KVA, SIL + 25 wt.% PVAc, SIL + 50 wt.% PVAc and SIL + 75 wt.% PVAc) were examined using DSC. During these studies, the samples were heated from 320 to 475 K and 300 to 475 K for the KVA- and PVAc-based compositions respectively, at a rate of 10 K/min. Thermograms presented in Figure 1 correspond to the fully amorphous samples obtained through the vitrification process. In both panels: A and B, one can observe up to three thermal events during each scan. Starting from the highest temperatures, the first is the endothermic peak that corresponds to the sample melting process (visible for neat SIL and compositions containing up to 50 wt.% KVA and 75 wt.% PVAc). Second is the exothermic peak that corresponds to the cold crystallization of the amorphous sample (visible for neat SIL and compositions containing up to 50 wt.% KVA and 75 wt.% PVA—marked with red arrows). Third, present in each sample is a single step-like thermal event that corresponds to the glass transition (marked with black arrows). It has been demonstrated by Baird et al. that when the phase separation occurs, two separate thermal events (glass transitions) should be visible in a DSC thermogram [52]. Furthermore, as was shown by Qian et al., a single T_g_ corresponds to the homogeneous mixing of the sample [53]. Therefore, our results might imply the homogeneity of the samples. However, it should be pointed out that this conclusion relies on the DSC analysis and the resolution of this method is low (approx. 30nm) [54]. Values of the T_g_ of neat amorphous SIL as well as KVA and PVAc polymers are equal to 331, 376 and 314 K, respectively, which is in a perfect agreement with the literature [8,49,55]. In the case of the binary mixtures containing SIL and 25, 50, and 75 wt.% of KVA, the T_g_ is equal to 342, 353 and 366 K, respectively, while for the binary mixtures containing 25, 50 and 75 wt.% of PVAc, the T_g_ is equal to 325, 320 and 316 K, respectively.

It should be noted that the T_g_ of the investigated KVA-based systems increases continuously, with an increasing polymer content. This behavior is common and is caused by the antiplasticization effect exerted by the polymer, with a T_g_ 46 K higher than of the neat drug. In contrast to this phenomenon, in the case of the PVAc-based systems, the T_g_ continuously decreases with an increasing amount of polymer, which can be explained by the plasticization effect exerted by the polymer, which has a T_g_ lower by 18 K than the neat drug. Aside from the discussed effects, the additional factor that could affect the sample T_g_ is the existence of specific interactions between the drug and the polymer. It is worth recalling that, if the plasticization or antiplasticization effect is dominant, the T_g_ of the mixtures should vary with polymer content in accordance with the Gordon–Taylor prediction, which is defined as follows:

(1) $$T_{g}=\frac{W_{1}T_{g1}+{\mathrm{KW}}_{2}T_{g2}}{W_{1}+{\mathrm{KW}}_{2}}$$

where the T_g_, T_g1_, T_g2_ are the glass transition temperatures of the drug-polymer mixture, the amorphous drug, and the polymer, respectively; W_1_ and W_2_ are the weight fraction of the drug and polymer and K is a parameter that can be calculated according to the formula:

(2) $$K\approx \frac{\Delta C_{p2}}{\Delta C_{p1}}$$

where ∆C_p1_ and ∆C_p2_ are the changes in heat capacity at T_g_ of drug and polymer, accordingly.

In Figure 1C,D, the experimentally obtained T_g_ values of the mixtures with different concentration of SIL and PVAc or KVA (presented as circles), have been compared with T_g_ values predicted from the Gordon–Taylor equation (Equation (1)) (marked as orange solid lines). In most cases, the drug-polymer mixtures show some deviation between the experimental and theoretical values, due to nonideal mixing. Stronger intermolecular interactions are usually preferred and result in a favorable exothermic mixing with increased configurational entropy [56]. It can be seen that the T_g_s of SIL-based amorphous dispersions are in a perfect agreement with those calculated by Equation (1). (see Figure 1C,D). This implies that the drug should be miscible and evenly dispersed within each polymer [7]. Moreover, it should be emphasized that highly miscible systems are found to be more resistant to the recrystallization process. Although the lack of deviation from the G-T prediction might suggests the absence of any specific drug-polymer interactions [7], it is not conclusive proof. Therefore, IR studies to determine whether intermolecular interactions are present in the ASDs were performed.

**Figure 2:**
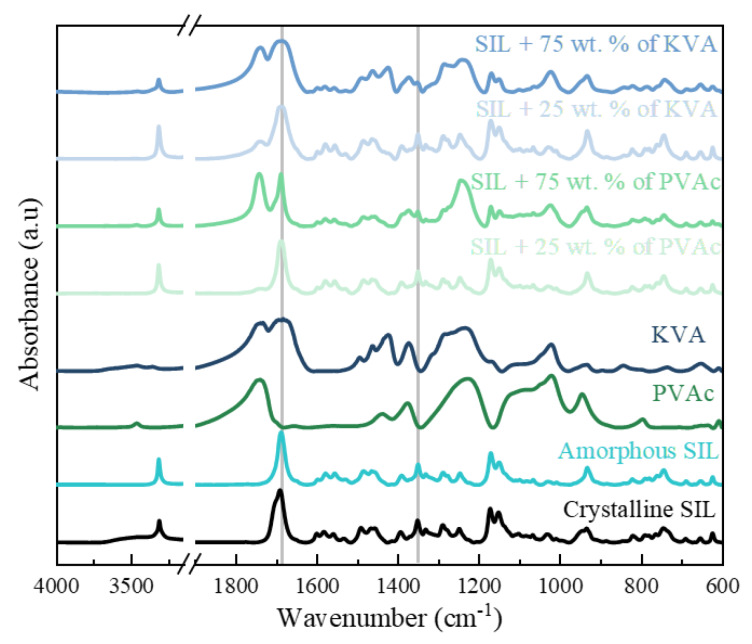
Experimental infrared spectra of crystalline, as well as amorphous, SIL and SIL-based amorphous solid dispersion (ASD) samples, which were prepared by melting the binary physical mixture at 468 K and then quenched. Grey lines at 1687 cm^−1^ and 1349 cm^−1^ correspond the to –C=O and –S=O peaks, respectively.

The key vibrations of SIL are: –N–H stretching at 3312 and 3315 cm^−1^, –C=O stretching at 1690 and 1687 cm^−1^, –S=O asymmetric stretching at 1351 and 1349 cm^−1^, and –S=O symmetric stretching at 1172 and 1170 cm^−1^ for the “as supplied”, crystalline and amorphous SIL, respectively [57]. The red shift of the –N–H band could be due to the breaking/weakening of the intramolecular H-bond that links the –N–H group to the ethoxy moiety in SIL. When mixed with PVAc, SIL in the amorphous phase did not show any considerable shifts of the above principal bands. The –C=O peak moved from 1687 to 1688 cm^−1^ only in the polymer-based samples. A similar behavior of the drug was noticed in the ASDs with KVA, where the –C=O band shifted to 1688 cm^−1^, while the peak of the –S=O asymmetric stretching moved from 1349 to 1351 cm^−1^; see Figure 2. It can therefore be concluded that no interactions between SIL and the polymers in ASDs can be detected by infrared spectroscopy. It is most likely, as SIL has only one H-bond donor (–N–H), also involved in an intramolecular H-bond, and is poorly accessible (steric hindrance) by the H-bond acceptor groups (–C=O) of the polymers. This is of a great importance, since the presence of interactions would be an additional factor affecting the physical stability [40,42,58,59,60].

Considering the experimental time frame, upon heating above the samples T_g_, only the neat polymers and the drug-polymer composition containing 75 wt.% of KVA do not exhibit an exothermic event corresponding to the recrystallization (see Figure 1 A,B). The presence of the SIL devitrification process in mixtures containing up to 50 wt.% of KVA and 75 wt.% of PVAc could be explained by its large tendency towards recrystallization, as the neat drug was classified as “moderately fragile” [49]. Focusing on the KVA-based mixtures, one can observe that the recrystallization process is inhibited by the increasing polymer content (see Figure 1B). This is a very common phenomenon seen in binary systems of a drug mixed with a high-T_g_ polymer and it has been associated with the deceleration of the molecular mobility [61]. In the case of SIL + 25 wt.% PVAc, the acceleration of the molecular mobility, exerted by the addition of a low-T_g_ polymer, decreased the temperature of the crystallization onset (see Figure 1A). This result is consistent with the data reported for the binary mixture of indomethacin and PEO, where the polymer addition accelerated the crystallization process [46]. However, as can be seen in Figure 1A, with a further increase in the concentration of this polymeric additive (PVAc), the onset of the SIL recrystallization process shifts towards higher temperatures. Furthermore, we performed additional analysis of the calorimetric data. We compared the recrystallization enthalpies (corrected for the drug content in each ASDs) to directly asses the inhibiting effect of both additives. The results are presented in Table 1. It can be seen that KVA seems to be a better stabilizer of amorphous SIL than PVAc. However, it should be pointed out that the amorphous drug does not recrystallize completely, regardless of the polymer used.

One can conclude, based on the calorimetric data, that above a certain concentration, both KVA and PVAc can improve the physical stability of the amorphous form of SIL. This improvement can be observed alongside two opposite effects, where, on one hand, one of the polymeric additives (in this case, KVA) increases the T_g_ of SIL (consistent with slowing down its molecular mobility) and onthe other hand, the other polymer, PVAc, decreases the T_g_ of SIL (consistent with accelerating its molecular mobility). Due to the fact that molecular dynamics have been frequently found to be a key factor affecting the physical stability of amorphous pharmaceuticals [62,63], in the following section of this work, we investigated the molecular mobility of the amorphous form of SIL-based ASD compositions via non-isothermal and isothermal dielectric spectroscopy.

### 3.2. Non-Isothermal Dielectric Measurements

**Figure 3:**
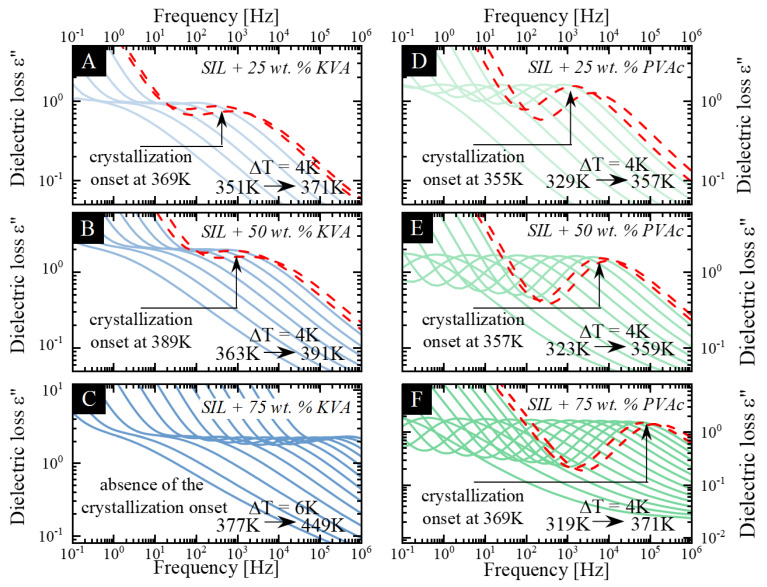
Dielectric spectra obtained during non-isothermal measurements. (**A**–**C**) presents the SIL + 25% KVA, SIL + 50% KVA and SIL + 75% KVA, respectively. (**D**–**F**) presents the SIL + 25% PVAc, SIL + 50% PVAc and SIL + 75% PVAc, respectively. Red dashed lines correspond to the recrystallization process.

In order to determine the molecular dynamics of each of the prepared sample, we performed a series of dielectric measurements in a wide frequency range. Since the molecular dynamics of the neat SIL have already been reported [49], the following section of this manuscript focuses mainly on its binary ASDs. The representative dielectric loss spectra of the SIL–KVA and SIL–PVAc compositions are shown in Figure 3A–C, and Figure 3D–F, respectively. Spectra presented in Figure 3 were recorded above the sample’s T_g_s. In all cases, they exhibit one loss peak that corresponds to the structural α relaxation and moves towards higher frequencies with increasing temperature.

**Figure 4:**
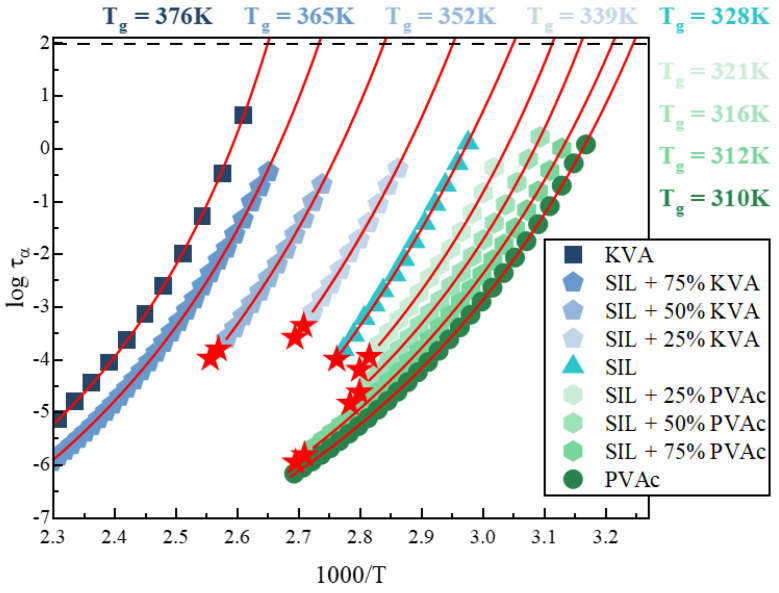
Relaxation map of binary mixtures of (from left): KVA (royal square), SIL + 75 wt.% KVA (cornflower pentagon), SIL + 50 wt.% KVA (baby blue pentagon), SIL + 25 wt.% KVA (celeste blue pentagon), SIL (aqua triangles), SIL + 75 wt.% KVA (cornflower pentagon), SIL + 75 wt.% KVA (cornflower pentagon), SIL + 75 wt.% KVA (cornflower pentagon) and PVAc (olive circles). Red stars indicate the crystallization onset. Temperature dependence of τ_α_ in the supercooled liquid was described by Vogel–Fulcher–Tamman (VFT) equations (red solid lines). Data regarding neat SIL were taken from [49].

From the analysis of loss spectra recorded in the supercooled liquid state, we determined the temperature dependencies of the α-relaxation times (τ_α_(T)) for all prepared systems (see Figure 4). In order to obtain the values of the relaxation time (τ_α_) at various temperatures, the experimental data were fitted using the Havriliak–Negami (HN) function (Equitation (3)): [64]

(3) $$\varepsilon^{*}\left(\omega \right)=\varepsilon_{\infty }+\frac{\Delta \varepsilon }{{\left[1+{\left({i\omega \tau }_{\mathrm{HN}}\right)}^{a}\right]}^{b}}+\frac{\sigma_{\mathrm{dc}}}{\varepsilon_{0}i\omega }$$

where ε_∞_ is high frequency limit permittivity, ε_0_ is the permittivity of vacuum, ∆ε is dielectric strength, ω is equal to 2πf, τ_HN_ is the HN relaxation time, a and b are parameters representing symmetric and asymmetric broadening of relaxation peak, σ_dc_ is the conductivity and ε_0_ is the dielectric permittivity of the vacuum. The values of τ_α_ were calculated, using the fitting parameters determined above, by means of the following formula:

(4) $$\tau_{\alpha }=\tau_{\mathrm{HN}}{\left[\sin\left(\frac{\pi a}{2+2b}\right)\right]}^{\frac{1}{a}}{\left[\sin\left(\frac{\pi \mathrm{ab}}{2+2b}\right)\right]}^{-\frac{1}{a}}$$

The relaxation times obtained from the described fitting procedure are presented in Figure 4. This figure clearly presents the acceleration or deceleration of the molecular mobility of the neat drug (triangles) in the presence of a polymeric additive, PVAc (hexagons) or KVA (pentagons) respectively.

The temperature evolution of the structural relaxation time in the supercooled liquid region usually shows a non-Arrhenius like behavior. Therefore, in order to parameterize it, the Vogel–Fulcher–Tamman (VFT) equation, defined as follows, was used [65,66,67]:

(5) $$\tau_{\alpha }\left(T\right)=\tau_{\infty }\exp\left(\frac{B}{T-T_{0}}\right)$$

where τ_∞_, B and T_0_ are the fitting parameters. It should be pointed out that the time scale for relaxation within the supercooled liquid increases as the temperature decreases and, considering the situation where crystallization does not occur, it becomes of the order of hundreds of seconds when the temperature enters the glass range. This internal relaxation time at large values (10^2^ s) causes the glass "transition" at T_g_ [68]. Therefore, in order to determine the T_g_ values, we employed the definition frequently used in dielectric studies: T_g_ = T(τ_α_ = 100s). Accordingly, from the extrapolation of the VFT fit to 100s, we subsequently estimated the T_g_ of: KVA, SIL + 75 wt.% KVA, SIL + 50 wt.% KVA, SIL + 25 wt.% KVA, SIL, SIL + 25 wt.% PVAc, SIL + 50 wt.% PVAc, SIL + 75 wt.% PVAc and PVAc as 377, 365, 352, 339 and 328 K [49], 321, 316, 312 and 310 K [47], respectively (see Figure 4). Considering the fact that the T_g_ is dependent on the heating rate and different heating rates were applied in both types (DSC vs. BDS) of experiments, the results from the dielectric study were in a very good agreement with the calorimetric data. Furthermore, a good agreement of the Tg values obtained from calorimetric as well as dielectric measurements was confirmed for the example of amorphous gambogic acid, based on the heating rate dependence of Tg calculated using the Elastically Collective Nonlinear Langevin Equation (ECNLE) theory [69].

Upon heating above the T_g_, at a certain temperature, one can observe a significant decrease in the intensity of the structural relaxation peak (visible on dielectric loss spectra—marked as the red dashed line in Figure 3), which corresponds to the decrease in the dielectric strength. Taking into account that the dielectric strength is proportional to the number of units (N), with a dipole moment (μ) involved in the structural relaxation (Δε ∼ Nμ^2^), and the fact that the number of reorienting dipoles, contributing to the α-relaxation, decreases as the crystallization process progresses [70,71], this drop reflects the onset of the recrystallization of the amorphous sample. It should be emphasized that the only sample that did not exhibit a tendency towards the recrystallization, during the time frame of the experiment, was the SIL + 75 wt.% KVA composition. The temperature of the crystallization onset (T_C_), the temperature at which the first spectrum reflecting the noticeable drop in the intensity was recorded, for the following samples: SIL + 50 wt.% KVA, SIL + 25 wt.% KVA, SIL, SIL + 25 wt.% PVAc, SIL + 50 wt.% PVAc and SIL + 75 wt.% PVAc, was equal to 389, 369 and 360 K [49], 355, 357 and 369 K, respectively. The differences between the BDS and DSC results can be explained by the different heating rates applied. Recently, Kołodziejczyk et al. [49] showed that SIL is very sensitive to the different analytical conditions.

It should be noted that all KVA-based systems were characterized by higher T_C_ values than that of neat SIL, see Figure 3. This would imply the superior stabilization properties of the KVA polymer in the binary mixtures with SIL, when compared to the effect exerted by PVAc. Moreover, based on the collected dielectric spectra the ASD systems containing up to 50 wt.% of PVAc are characterized by the lower temperature of the crystallization onset than amorphous SIL (see Figure 3). This would support the hypothesis that the acceleration of the molecular dynamics of the system would increase its tendency towards recrystallization [46]. On the other hand, it should be emphasized that with each subsequently measured concentrations of the polymer additive, the T_C_ shifts towards higher temperatures, which indicates an improvement of the physical stability. In addition to the T_C_, attention should also be paid to the characteristic relaxation time of the sample at the onset of crystallization. Focusing on the SIL + KVA mixtures (see Figure 3), simultaneously with the deceleration of the molecular dynamics of the system (in the presence of an increasing polymer content—up to the 50 wt.% of KVA) the recrystallization starts at a longer τ_α_ than in neat, amorphous SIL [49]. This would imply that the molecular motion associated with the primary relaxation process of the binary mixture does not have to be as fast as in the neat drug in order to observe the onset of the recrystallization process. On the other hand, the recrystallization in the PVAc-based systems occurs at shorter relaxation times than in the neat drug (except the SIL+ 25 wt.% PVAc, where the τ_α_ at the onset of crystallization seems to be nearly the same for the neat SIL [49]). This indicates that the molecular mobility of the binary mixtures based on α–relaxation, at the onset of the recrystallization process, is significantly faster than in the neat drug (up to nearly two orders of magnitude in the case of SIL + 75 wt.% PVAc). A similar phenomenon has been shown for other binary systems [18,47].

**Figure 5:**
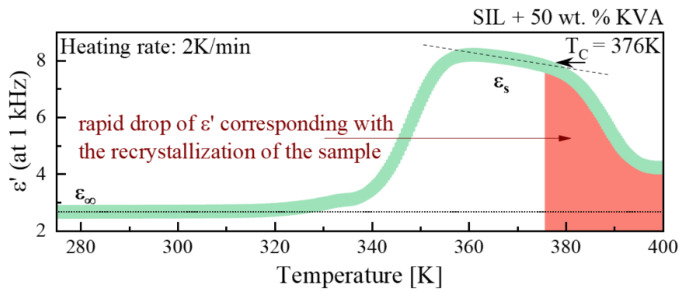
The evolution of the dielectric constant ε′ at 1 kHz, as measured for SIL + 50 wt.% KVA upon heating at a 2 K/min rate. Red dashed area corresponds to the sample recrystallization process.

Next, in order to more precisely evaluate the crystallization tendencies, we performed additional measurements based on a single frequency (1 kHz), with a heating rate of 2 K/min. Figure 5 demonstrates the changes in the real part of complex dielectric permittivity (registered at 1 kHz) in the representative example, SIL + 50 wt.% PVAc ASD. Starting from the low temperature measurements, ε’ takes the value that corresponds to the high-frequency permittivity limit (ε_∞_). Upon further heating, in the vicinity of 340 K, a characteristic step-like change of the ε′ value, from the ε_∞_ to the static dielectric permittivity (ε_s_), associated with the primary (α) relaxation process, is visible. By calculating the difference between the values of ε_s_ and ε_∞_, one can determine the dielectric strength (Δε = ε_s_ - ε_∞_). It is worth noting that the Δε of the α-relaxation should decrease with temperature as a consequence of the thermal agitation, bringing in disorder against the electric field orientation (according to the Langevin formula by a term proportional to 1/T). Nevertheless, a further increase in the temperature (> 375 K) resulted in a rapid drop of the dielectric permittivity (Figure 5). Similar to the measurements performed in the wide frequency range, this drop corresponds to the recrystallization process.

**Figure 6:**
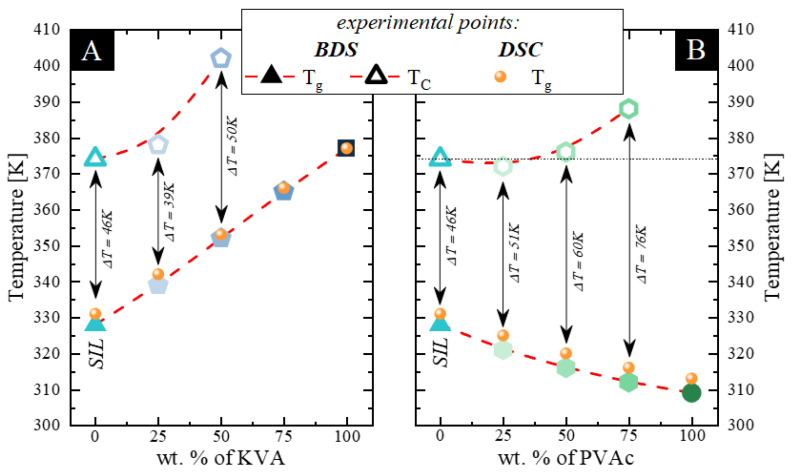
Experimental data determined by broadband dielectric spectroscopy (BDS). (**A**,**B**) present the temperatures of the crystallization onset (empty symbols) as well as the glass transition temperatures (full symbols) of SIL–KVA and SIL–PVAc ASDs, respectively. Red dashed lines are present to help visualize the trend. Data regarding the neat SIL was taken from [49].

The first temperature at which a noticeable decrease in the dielectric strength was recorded, which corresponds to the T_C_, for the following samples: SIL + 50 wt.% KVA, SIL + 25 wt.% KVA, SIL, SIL + 25 wt.% PVAc, SIL + 50 wt.% PVAc and SIL + 75 wt.% PVAc was measured as 402, 378, 374, 372, 376 and 388 K, respectively (see Figure 6 A,B).

It should be pointed out, that at 25 and 50 wt.% of KVA, the onset of the SIL cold crystallization shifts towards higher temperatures. Moreover, the sample containing 75 wt.% of this polymer fully inhibited the SIL tendency towards recrystallization (within the timeframe of the experiment). In the case of the PVAc-based systems, all samples began to crystallize during the measurement. It is worth emphasizing that the recrystallization process in the SIL + 25 wt.% PVAc sample occurs at a lower temperature than in the neat, amorphous drug (see Figure 6B). A further increase in the polymer content resulted in the shift of the T_C_ above the SIL crystallization onset. This indicates that above a certain concentration, PVAc stops accelerating the recrystallization process and starts to inhibit it. This can be attributed to the fact that at the high polymer concentration, the steric hindrance that slows or even prevents the formation of the crystals can occur. These results are in a good agreement with the calorimetric data, despite the much slower heating rate of 2 K/min.

Furthermore, we determined the thermal stability factor (the difference between T_g_ and T_C_), which is one of the measures of glass stability [72,73]. This factor takes into account the T_g_, which is important, considering both plasticization and antiplasticization effect exerted by the excipients used. Focusing on the KVA-based systems, one can observe that despite the fact that 25 wt.% addition of KVA shifts the onset of the SIL cold crystallization towards higher temperatures, this system is actually characterized by a lower thermal stability factor than the neat drug (see Figure 6A). Considering the non-isothermal measurements performed in this work, the enhancement of the physical stability exerted by this specific amount of the antiplasticizing polymer is, therefore, only apparent. The explanation of this result is related to the fact that although the temperature range in which the sample is stable in the supercooled liquid state (above T_g_) was shifted towards higher values, its actual range is narrower. Nevertheless, a further increase in the polymer content resulted in a higher thermal stability factor than in the case of amorphous SIL. Additionally, for the PVAc-based systems, one can observe that each subsequent concentration of the plasticizer used has a higher thermal stability factor than the neat drug (see Figure 6B). This result, although unexpected, because 25 wt.% addition of PVAc shifts the onset of the cold crystallization towards lower temperatures, has a similar explanation to the phenomenon observed for the sample with 25 wt.% of KVA. Namely, the temperature range, in the supercooled liquid state, in which the sample is stable, was shifted towards lower values, but its actual range is broader. Due to the above, the improvement in the physical stability should be considered, with regard to either: i) the specific temperature at which the samples are stored, or ii) the conditions at which the molecular mobility of the samples is the same (relaxation time is constant). The latter is interesting, due to the significant changes in the molecular mobility (both its acceleration and deceleration) caused by the excipients; see Figure 4.

The current studies indicate that regardless of the additive used, whether showing an antiplasticizing (KVA) or plasticizing (PVAc) effect, the physical stability improves as the polymer concentration increases. However, due to the lack of a clear answer on whether a small amount (up to 25 wt.%) of the polymer improves the physical stability of the amorphous API, in the next section of this work, two different conditions (at a constant temperature and a constant relaxation time) at which the physical stability of the examined systems can be evaluated, was used in crystallization studies. On one hand, the samples can be stored at isothermal conditions, regardless of their τ_α_; on the other hand, due to the deceleration or acceleration of the molecular dynamics (with regard to additive used), it is worth considering the isochronal conditions (choosing the temperature so that the τ_α_ of the different samples is constant).

### 3.3. Isothermal and Isochronal Dielectric Measurements

In order to get an insight into the effect of the polymeric additives used on the physical stability of amorphous SIL, we focused on two approaches. Namely, the neat amorphous SIL as well as its binary ASDs containing KVA or PVAc polymer, were evaluated, utilizing BDS, at: i) isothermal conditions (at the same temperature T = 353 K), as well as ii) isochronal conditions (at temperatures at which the samples exhibit the same relaxation time τ_α_ = 1.5 ms). To help visualize the conditions of the time-dependent dielectric measurements performed, all temperatures were marked on the DSC thermogram provided in panel A of the corresponding Figures.

**Figure 7:**
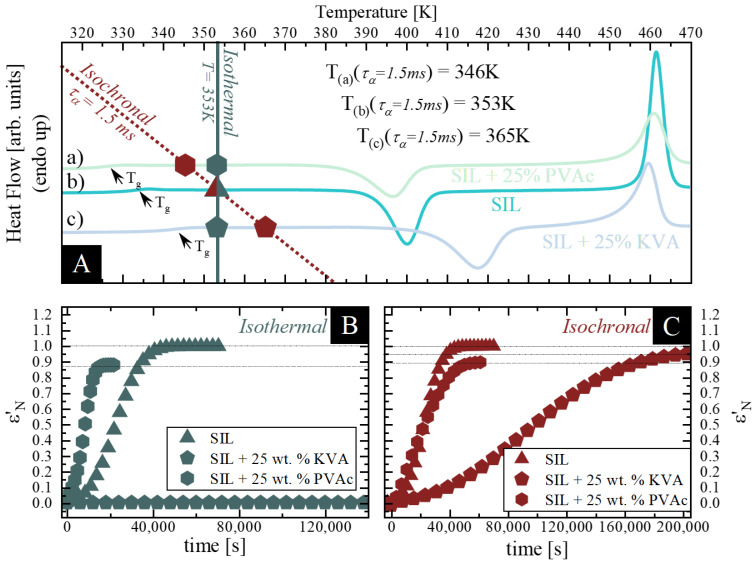
**A** presents the thermograms of a) SIL + 25 wt.% PVAc (sage); b) SIL (aqua) and c) SIL + 25 wt.% KVA (celeste blue). Wine hexagon, triangle and pentagon correspond to the temperatures at which the relaxation time of SIL + 25 wt.% PVAc (T = 346 K), SIL (T = 353 K) and SIL + 25 wt.% KVA (T = 365 K), respectively, is equal to τ_α_ = 1.5ms. Slate hexagon, triangle and pentagon correspond to the points of the SIL + 25 wt.% PVAc; SIL and SIL + 25 wt.% KVA samples at 353 K. **B** shows the isothermal crystallization of SIL, SIL + 25 wt.% PVAc and SIL + 25 wt.% KVA as slate triangle, hexagon and pentagon, respectively. **C** shows the isochronal (τ_α_ = 1.5ms) crystallization of SIL (at T = 353K), SIL + 25 wt.% PVAc (at T = 346 K) and SIL + 25 wt.% KVA (at T = 365 K), as wine triangle, hexagon and pentagon, respectively. Data regarding the neat SIL were taken from [49].

As stated in the previous section, the crystallization process during dielectric studies is manifested as a decrease of the dielectric strength. This can be observed in the real part of the dielectric permittivity (ε’) (see Figure 5), as well as in the imaginary part of the dielectric permittivity (ε”) (see Figure 3). By tracking the time-evolution of dielectric strength, one can easily monitor the kinetics of a crystallization process. Therefore, the following analysis is based on the ε’ data. At the beginning, we normalized the real part of dielectric permittivity, which can be expressed as follows [5]:

(6) $$\varepsilon_{N}^{\text{’}}\left(t\right)=\frac{\varepsilon \text{′}\left(0\right)-\varepsilon \text{′}\left(t\right)}{\varepsilon \text{′}\left(0\right)-\epsilon^{\prime }\left(\infty \right)}$$

where *ε′(0)* is the initial static dielectric permittivity, *ε′(t)* is the value at time *t*, and *ε′(∞)* is the high-frequency permittivity limit. Figure 7B,C present the plots of ε’_N_(t) as a function of time for each sample evaluated at given conditions. Beginning with the isothermal measurements (at 353 K) of the samples with the low concentration of the polymeric additive (25 wt.% of KVA and PVAc) and their comparison to neat SIL (data taken from [49]), one can observe the following: i) SIL + 25 wt.% KVA does not exhibit any tendency towards recrystallization up to 36h under these conditions (see slate pentagons in Figure 7B) and ii) SIL + 25 wt.% PVAc starts to recrystallize noticeably sooner than neat SIL [49] (see slate hexagons and triangles, respectively, in Figure 7B). These results would imply that the 25 wt.% addition of KVA inhibits the recrystallization of neat SIL, whilst the 25 wt.% addition of PVAc accelerates its devitrification process.

**Figure 8:**
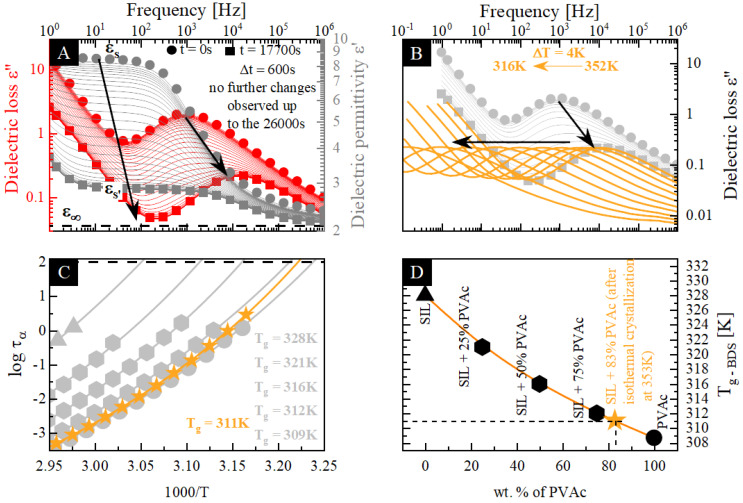
(**A**) presents dielectric spectra obtained during isothermal measurements (at 353 K) performed on the fully amorphous sample (SIL + 25% PVAc). (**B**) presents additional measurements performed on the partially amorphous sample, after its recrystallization at 353 K. Grey lines correspond to the isothermal crystallization while the orange lines correspond to the non-isothermal measurements performed during cooling from the temperature of performed isothermal measurement to 316 K. (**C**) shows the temperature dependence of the relaxation times of the fully amorphous samples (grey triangles), as well as the samples after isothermal crystallization (orange stars). Temperature dependence of τ_α_ in the supercooled liquid was described by the VFT equations (grey and orange solid lines). (**D**) shows a concentration dependence of the glass transition temperatures of the SIL-based ASD systems, determined by BDS. Grey triangle, circle and hexagons are assigned to the neat amorphous SIL, PVAc and its binary mixtures. Orange star refers to the concentrations determined via isothermal measurements performed at 353 K.

These results, similar to the non-isothermal BDS measurements presented in the previous section, are consistent with the literature, whereby by accelerating molecular mobility of the system one would also accelerate the recrystallization process [46]. It has to be pointed out, however, that when the recrystallization process ceased (no further changes in the molecular dynamic behavior, either in its intensity and/or shifts of the relaxation time, were observed over a significant period of time), the value of the static dielectric permittivity was still significantly higher than its high-frequency permittivity limit (see Figure 8A), which corresponds to the residual relaxation process remaining after the recrystallization. This phenomenon has been well described for an antiandrogen drug, flutamide [8]. During the recrystallization of the excess amount of the drug, due to the changes in concentration (decreasing amount of amorphous drug fraction), one could observe the apparent increase in the polymer concentration, which in turn affected the position of the relaxation process peak. Therefore, considering the binary systems of a drug and a polymer, this remaining process can be ascribed to either: i) segmental or secondary relaxation originating from the residual amorphous polymer, which remained after the recrystallization of the whole drug fraction from the mixture, as it was already observed in the case of other drug-polymer mixtures, ii) the primary relaxation of a different than the initial drug concentration, after the recrystallization of the excess amount of the drug from the supersaturated API-polymer mixture [4,8,47,55,74,75]. Focusing on the former, when the whole amount of the amorphous SIL recrystallizes in the binary mixture; the residual relaxation process, related to the remaining amorphous polymer, should still be visible. Furthermore, complete recrystallization of one of the components would imply their mutual immiscibility. The second possibility, in contrast to the above, is associated with reaching the solubility limit of the drug within the polymer matrix at a certain temperature. Namely, the recrystallization observed concerns only the excess amount of the drug from its supersaturated solution. Therefore, when the recrystallization ceases, a system with a different than the initial concentration (saturated solution), still contributes to the static dielectric permittivity. Despite these two contradictory explanations, both seem probable. However, it should be highlighted that the T_g_ of PVAc is 310 K when measured via BDS, which means that at the temperature of the recrystallization studies performed at 353 K, its segmental relaxation should be well visible in the experimental frequency range. Accordingly, one can determine the relaxation time of neat amorphous polymer at a given temperature (i.e., 353 K) and compare it with the one determined for the process remaining after the sample crystallization. In this case, the values of τ_α_ differ (data not shown) which imply that this remaining process does not correspond solely to the segmental relaxation of the neat polymer. Furthermore, we performed additional measurements to validate if the observed phenomenon in fact corresponds to the SIL’s solubility limit in the chosen polymer matrix. As presented in Figure 8B, when the recrystallization process ceased, we remeasured the sample during cooling in order to determine τ_α’_(T), where the α’ refers to the relaxation process still observed after recrystallization. Further investigations focused mainly on the determination of the T_g_ of the sample obtained after isothermal recrystallization. This can be done by the extrapolation of its VFT fit to 100s. Once the T_g_ of this system (saturated solution) was determined (see Figure 8C), its composition can be easily established. It can be done by comparing the obtained T_g_s value to those experimentally determined by plotting the concentration dependence of the T_g_s. It can be seen in Figure 8D that the saturated composition of SIL in the SIL-PVAc system at 353 K was 17 wt.% of SIL. However, it must be pointed out that due to the small changes in the T_g_ at higher polymer concentrations (i.e., ΔT_g_ = 3 K between SIL + 75 wt.% PVAc and the neat PVAc), the result obtained might be burdened with some uncertainties. As such, without additional confirmation, it should be treated as qualitative and not quantitative. Nevertheless, the determination of drug-polymer solubility is a critical parameter when considering the optimal ratio between the drug and polymer, in order to ensure thermodynamic stability [38,39,40]. From the practical point of view, the use of a plasticizing compound as the inhibitor of crystallization (PVAc) would not be very effective in the presented case, due to the amount of additive required to obtain a stable composition.

Next, we performed isochronal measurements (at τ_α_ = 1.5ms). One can observe in Figure 7C that, similar as in the case of the recrystallization of SIL + 25 wt.% PVAc at 353 K, both SIL + 25 wt.% KVA and PVAc samples did not recrystallize fully. The noteworthy observation is that both samples (SIL + 25 wt.% KVA and PVAc) start to recrystallize after nearly the same time as the neat SIL (see Figure 7C). Moreover, the crystallization process of both SIL + 25 wt.% PVAc, as well as the neat SIL at isochronal conditions, is very similar (both the induction times as well as the time when the recrystallization ceases—see wine hexagons and triangles, respectively, in Figure 7C). This result could be explained by the fact that either: i) the molecular dynamics is the crucial factor affecting physical stability of amorphous API, which would be in agreement with the literature [62,63], or ii) this specific amount of PVAc does not affect the SIL crystallization kinetics at these specific conditions. Unlikely as it is, in the discussed case, authors lean towards the latter explanation, considering that the former would not apply to the KVA-based compositions. Based only on the results for neat SIL and the SIL + 25 wt.% of PVAc mixture, one can observe that when the molecular dynamics of both systems are in an agreement (the same relaxation time), their tendency towards recrystallization (kinetics of crystallization) is characterized by the matching induction time and the time when the recrystallization ceases. This would indicate that the molecular mobility, and not the temperature, is the key factor controlling this process. However, this is not true when one considers the SIL + 25 wt.% of KVA mixture. Despite the similar induction time of the recrystallization process, its overall kinetics are slower when compared to the neat drug (see Figure 7C). This does not support the hypothesis that molecular mobility is the key factor determining the physical stability and confirms the superior stabilization properties of KVA in the binary mixtures with SIL. Furthermore, it should be mentioned that both samples did not recrystallize completely. After performing additional analysis, as described in the previous section, we were able to determine the solubility limits of SIL in both used polymers, 10 wt.% of SIL in the PVAc matrix at 346 K and 6 wt.% of SIL in the KVA matrix at 365 K. Based on the results obtained and due to the fact that solubility increases with increasing temperature, one can deduce that PVAc is able to dissolve a higher amount of SIL than KVA. However, more detailed studies in this regard are required.

Based on the results presented above, one can conclude that the PVAc addition accelerates the recrystallization of amorphous SIL, while the KVA addition inhibits it, when stored at the same temperature condition. On the other hand, when stored at the isochronal conditions, KVA slows down the recrystallization process of the neat drug, while PVAc no longer accelerates it. Furthermore, it seems that at this exact concentration and these conditions, PVAc does not affect the kinetics of the devitrification process when compared to the neat SIL. It is worth considering whether the acceleration of the cold crystallization (or a neutral effect, depending on the storage conditions) will proceed with an increased polymer content or whether the opposite will happen, an increased concentration of PVAc will improve the physical stability of amorphous SIL, as indicated throughout the non-isothermal measurements.

**Figure 9:**
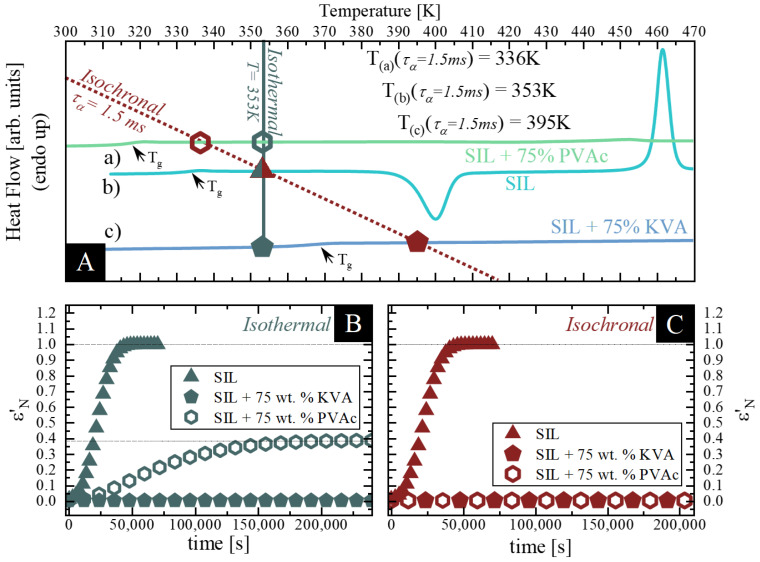
(**A**) presents the thermograms of a) SIL + 75 wt.% PVAc (emerald); b) SIL (aqua) and c) SIL + 75 wt.% KVA (cornflower). Wine hexagon, triangle and pentagon correspond to the temperatures at which the relaxation time of the SIL + 75 wt.% PVAc (T = 336K); SIL (T = 353K) and SIL + 75 wt.% KVA (T = 395K), respectively, is equal to τ_α_ = 1.5 ms. Slate hexagon, triangle and pentagon correspond to the points of the SIL + 75 wt.% PVAc; SIL and SIL + 75 wt.% KVA samples at 353 K. (**B**) shows the isothermal crystallization of SIL, SIL + 75 wt.% PVAc and SIL + 75 wt.% KVA, slate triangle, hexagon and pentagon, respectively. Panel C shows the isochronal (τ_α_ = 1.5ms) crystallization of SIL (at T = 353 K); SIL + 75 wt.% PVAc (at T = 336 K) and SIL + 75 wt.% KVA (at T = 395 K) as wine triangle, hexagon and pentagon respectively. Data regarding neat SIL was taken from [49]

As a next step, the samples comprising the polymer as the major component, SIL + 75 wt.% KVA and PVAc, were examined. As expected, a more pronounced antiplasticizing and plasticizing effect resulted in significant differences between the temperatures at which the samples exhibit the same relaxation time (τ_α_ = 1.5ms—see Figure 9A). This difference is equal to 59 K (336 K for SIL + 75 wt.% PVAc and 395 K for SIL + 75 wt.% KVA). Furthermore, focusing on the isothermal measurements, it should be pointed out that due to the significant antiplasticizing effect in the SIL + 75 wt.% KVA sample, the temperature of performed crystallization studies (353 K) is below its glass transition (T_g-BDS_ = 365 K). Therefore, the measuring procedure had to be altered. Therefore, immediately after vitrification, a single dielectric spectrum was collected above sample’s T_g_ (at 383 K). Next, the sample was cooled down to 353 K to perform the actual measurement. Finally, after a certain time of storage (67 h), a single dielectric spectrum, above the T_g_ of the sample, was remeasured to compare its dielectric strength. It is worth emphasizing that despite these additional steps during the measurement, which involved a temperature that could have induce recrystallization, SIL + 75 wt.% KVA did not reveal any tendency towards recrystallization at this temperature (see Figure 9B).

The 75 wt.% PVAc sample, in contrast to that comprising 25 wt.% PVAc, showed a deceleration of the recrystallization process of amorphous SIL. This phenomenon confirms that at a certain concentration, PVAc will cease to accelerate the SIL recrystallization process and start to inhibit it. Furthermore, it should be mentioned that this sample did not recrystallize completely. Moreover, because the sample containing 25 wt.% of the API still recrystallizes while stored at 353 K, one can conclude that the solubility of the SIL in the PVAc matrix at this temperature is lower than 25 wt.% (the exact concentration was not determined).

Finally, during the measurements at isochronal conditions both samples (SIL + 75 wt.% KVA and SIL + 75 wt.% PVAc) did not exhibit any tendency towards devitrification up to 58 h at the conditions used. Furthermore, as the amount of PVAc increased in the system, it no longer had a neutral effect on the SIL kinetics of crystallization, which was observed for the 25 wt.% PVAc content, while stored at isochronal conditions. One can conclude, based on the dielectric data, that an addition of either 75 wt.% KVA or PVAc is able to improve the physical stability of the amorphous form of SIL. This is an interesting result, confirming that there is a well-defined concentration at which PVAc no longer decreases the physical stability of the amorphous drug.

## 4. Conclusions

The main purpose of this paper was to evaluate the impact of both high- and low-T_g_ polymer additives on the physical stability of the amorphous SIL. The presented work shows that despite the significant changes in the sample’s molecular mobility, exerted by the antiplasticizing (KVA) and plasticizing (PVAc) polymers, both of these excipients are able to effectively stabilize amorphous form of the investigated API. Considering the drug-polymer concentrations, far above the solubility limits, the measurements at either: isothermal (353 K) or isochronal (τ_α_ = 1.5ms) conditions, indicated that KVA-based samples were characterized by a better physical stability in comparison to the PVAc-based systems. Furthermore, the results indicate that the 25 wt.% addition of PVAc accelerates the recrystallization of the amorphous SIL when stored at the same temperature (353 K). Based on the isochronal measurements (at τ_α_ = 1.5ms), the 25 wt.% addition of PVAc (which resulted in an acceleration of the molecular dynamics of SIL) did not affect the kinetics of crystallization of the neat SIL. However, above a certain concentration, the PVAc started to increase the SIL physical stability (as shown for the SIL + 75wt.% PVAc sample). Therefore, KVA (a high-T_g_ polymer) proved to be a more efficient inhibitor of SIL’s recrystallization, when compared to PVAc (a low-T_g_ polymer). Nevertheless, based on the presented results, one can conclude that the ability of PVAc to dissolve amorphous SIL is superior in comparison to KVA.

## Figures and Tables

**Table 1:** The recrystallization enthalpies (corrected for the drug content in each system) of amorphous SIL and SIL ASDs.

	100 wt.% of Active Pharmaceutical Ingredients (API)	75 wt.% of API	50 wt.% of API	25 wt.% of API
SIL + PVAc	70.8 ± 0.1 J/g	69.1 ± 0.1 J/g	69.0 ± 0.1 J/g	8.8 ± 0.1 J/g
SIL + KVA	70.8 ± 0.1 J/g	66.8 ± 0.1 J/g	5.2 ± 0.1 J/g	X
